# Modern Views of Malaria. I

**Published:** 1894-04-14

**Authors:** 


### Modern Views of Malaria.—I.

There are few subjects more interesting than the discovery that the varieties of ague are due to an amœba-like organism in the blood, and now that Laveran's work on the subject has been translated into English by the Sydenham Society, it is probable that the discovery will receive in this country the attention which it deserves.

In the introductory chapter the author gives an account of the various crude and speculative views which have been previously held as to the cause of malaria, and then he proceeds to give a description of the post-mortem appearances found in the bodies of those who have died of malaria, and he rightly dwells upon the hæmorrhages, especially under serous membranes, which are frequently seen.

The chief interest, however, centres in the blood. The parasite of malaria is seen in the blood in four forms—1, spherical bodies; 2, flagella; 3, crescent-shaped bodies; 4, segmented and rose-shaped bodies.

Spherical bodies are the most common form, and Laveran found them in the blood in 389 observations out of 432. They often show amœboid movements. They are hyaline, colourless, and transparent, varying from being considerably smaller than a red corpuscle to about twice its size. Usually granular pigmented bodies situated near the periphery are present, and they may show a rapid vibratory movement. These amœba-like organisims live at the expense of the red blood corpuscles, hence the fewer of the latter the more of the former are seen. These parasites attack the red blood corpuscles, and the result is that the latter become swollen, and their characteristic colour and shape disappear.

*Flagella.*—When a preparation of blood is examined it is sometimes found that the spherical bodies have flagella attached to them. These move very quickly, and in so doing create a considerable disturbance among the blood corpuscles in their neighbourhood. These flagella, of which one, two, three, or four may be seen attached to a single spherical body, are very transparent, and are not easy to see when they are at rest; they may become detached from their spherical bodies, and they go away free in the blood. At the free end of each flagellum there is often a little pyriform swelling. The flagella probably represent a stage in the development of the malarial parasite, and they ought not to be looked upon as pseudopodic, for they separate so readily and move away quite independently.

*Crescent-shaped Bodies.*—These are more or less cylindrical, sometimes pointed at their extremities, and usually bent so as to form a crescent. They are transparent and colourless, except towards the middle, where some pigment graunles may be found. The length of these crescent-shaped bodies is about the diameter of a red blood corpuscle. A very fine line is usually seen in the concavity, and this joins the two horns.

*Rose-shaped Bodies.*—These are spherical, pigmented in the centre and regularly segmented so as to give the appearance of a rose. They are not nearly so often found in the blood as the other three forms, and probably merely represent a developmental change of them.

The parasites in ague are more frequent in the blood either immediately before, during, or just after an attack. In certain cachetic patients who have become very tolerant of these hæmatozoa, they may be found during the whole of the period between the paroxysms. There are great individual variations as to the rapidity with which they disappear when the patient is treated with quinine. Out of 432 cases examined, spherical bodies alone were found 266 times, crescent-shaped bodies alone 43 times, spherical and crescent-shaped bodies 31 times, spherical bodies and flagella 59 times, and spherical and crescent-shaped bodies and flagella 33 times. The spherical bodies alone, or associated with other elements, have been found 389 times out of 432. The crescent-shaped bodies alone, or associated with other forms, 107 times. The flagella, always associated with spherical elements, have been seen in 92 patients. In nearly all the cases in which the crescent-shaped bodies have been observed the patients have had the malarial fever some time.

The probable history is this: the embryonic stage of the parasite is that the minute hyaline bodies which may be seen in the blood in ague grow to be the spherical bodies, and that these live on the red blood corpuscles and take in their pigment. After a time flagella develope, and are cast off free. What becomes of them is not known, but somehow the crescent and rose-shaped bodies enter into the cycle, which begins again at the minute hyaline bodies. Thus we have an amœba-like parasite living in the blood, feeding on the red corpuscles, and undergoing a cycle of changes in development similar to those undergone by many parasites.

In order to see the parasites the blood of the finger which has been previously dried is pricked and a very thin cover glass preparation is made. There is no need to seal it unless examination with a very high power is needed; nor is it necessary to take minute precautions against the introduction of foreign bodies, but it is very important to have the preparation sufficiently thin, and consequently as so little blood is examined each time to make repeated examination of several preparations before it is definitely concluded that no parasites are present. If it is a warm day a hot stage is not necessary, and a power of 400 diameters is sufficient. Permanent preparations may be made, but no mounting fluid should be used, for it renders the parasites particularly transparent. They may be double stained with methyl blue and eosine.

Up to the present time all attempts either to cultivate the parasite on artificial media, or to produce malaria in the lower animals by injections, have failed; still its appearance is so characteristic that there can be but little doubt that it is the cause of the malady, just as there is no doubt that the filaria is the cause of the disease with which it is associated. It is interesting to notice in confirmation of the views here expressed that many observers have found parasites in the blood of the lower animals in various diseases, thus they have been met with in frogs, rats, and birds. Experts are at present divided upon the question of whether the different forms of ague are caused by different species of this amœba-like parasite. Some think that there is but one parasite causing ague, whilst others hold that there are at least three species, viz., (a) the hæmatozoon of the tertian; (b) the hæmatozoon of the quartan; (c) the hæmatozoon of the irregular forms. Laveran inclines to the view that there is only one polymorphic hæmatozoon.

The form in which the parasite is found in the external world is not known, but it probably enters the body by means of drinking water, and this accords with the well-known fact that it is not met with at an altitude, but only in low-lying, badly-drained places where there is no fall for the water. It is a disease of the country, not of towns, and is especially liable to attack those who work hard in the marshy districts. Residence for many generations confers partial immunity. Blacks rarely suffer.

